# Trematode infection buffers heat stress in blue mussels *Mytilus edulis*: The role of heat shock proteins

**DOI:** 10.1111/1365-2656.70220

**Published:** 2026-01-26

**Authors:** Annika Greve, Jesper G. Sørensen, Mikael K. Sejr, Jakob Thyrring

**Affiliations:** ^1^ Department of Ecoscience, Marine Ecology Aarhus University Aarhus C Denmark; ^2^ Department of Biology, Section for Genetics, Ecology and Evolution Aarhus University Aarhus C Denmark

**Keywords:** bivalve, environmental stressor, heat shock response, host–parasite interaction, intertidal ecology, marine invertebrate, thermal resilience, trematode infection

## Abstract

1. The influence of parasite infection on host thermal tolerance remains poorly understood. To address this, we investigated how infection with the trematode *Himasthla elongata* affects survival and heat shock protein expression in the blue mussel *Mytilus edulis* following repeated exposure to heat stress in a simulated intertidal environment.
2. Two groups of mussels with experimentally induced low (55.3 ± 35.6 metacercariae per mussel) and high (148.6 ± 78.2 metacercariae per mussel) infection levels were exposed to air (31°C, 33°C or 35°C) for 2 h over 10 days to simulate a tidal cycle. Survival was assessed daily. In addition, the mRNA expression level of three heat shock genes (*hsp24*, *hsp70* and *hsp90) was assessed in mussels exposed to 17*°C *and 33*°C for 2 h over a three‐day period. Dissection confirmed clear differences in infection levels between groups.
3. Survival decreased significantly with increasing air temperature, but in the 35°C treatment, mussels with high infection levels exhibited a near‐significant increase in survival. Expression of *hsp24, hsp70* and *hsp90* increased with rising air temperatures, and high infection levels significantly upregulated *hsp90*.
4. Although trematode infection did not significantly increase survival, our results suggest that trematode infection can protect against thermal stress by upregulating specific heat shock proteins in *M. edulis*. The *hsp* responses point to a parasite‐induced tolerance mechanism, potentially through stress priming or frontloading, and highlight an overlooked role of parasitism in mediating thermal resilience in intertidal ecosystems.

## Graphical abstract

The study shows that parasite infection changes heat shock protein expression and can increase heat tolerance in blue mussels. By separating parasite and temperature effects, the results suggest that infection may improve survival during heat stress, highlighting important parasite–host interactions for climate change resilience.

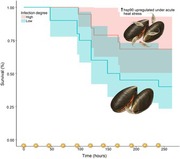

## 1 INTRODUCTION

Climate change has increased the frequency, intensity and severity of heat waves since the 1900s (Wang et al., 2024), with widespread effects on species and ecosystems (Arif et al., 2024). For example, billions of marine invertebrates perished during the 2021 Northwest Pacific heatwave, where air temperatures exceeded 49°C (White et al., 2023). Considering many invertebrates lack the ability to regulate their internal body temperature, the unpredictability, frequency and severity of extreme temperature events pose a greater threat to them than gradual increases in average temperatures (Renault et al., 2022). Thus, it is essential to understand how invertebrates cope with short‐term temperature extremes, particularly when these coincide with other stressors.

The interactions among co‐occurring abiotic (e.g. pH, hypoxia, salinity) (Earhart et al., 2022; Minuti et al., 2021; Nielsen et al., 2021; Pörtner, 2012; Thyrring et al., 2023; Venâncio et al., 2023) and biotic stressors (e.g. species interactions) can have complex effects on organismal physiology and influence the susceptibility of natural populations to thermal stress (Glibert et al., 2022). For instance, reduced salinity in coastal waters exacerbates the vulnerability of blue mussels (*Mytilus edulis*) to aerial heat stress (Barrett et al., 2022; Nielsen et al., 2021), while combined exposure to high air temperatures and low pH enhances thermal tolerance in some crustaceans (Paganini et al., 2014). Species interactions can also influence thermal tolerance and stress resilience. In Australia, the symbiotic relationship between corals and algae has been shown to enhance corals' heat‐stress tolerance (Berkelmans & Van Oppen, 2006), and in some mussel species, host shell‐boring symbiotic endolithic microbes can reduce thermal stress by altering shell porosity and albedo (Gehman & Harley, 2019; Lourenço et al., 2017; Zardi et al., 2016, 2021). In other host–parasite systems, decreased thermal tolerance has been attributed to parasitic infection (Greenspan et al., 2017). Clearly, responses to parasitic infection are not consistent among host–parasite systems, highlighting that the direction and magnitude of biological responses can vary widely among species and environmental settings, complicating efforts to predict ecosystem responses under future climate scenarios. Still, parasites remain frequently overlooked in studies of animal performance, despite their potential to influence responses to environmental stressors (Chrétien et al., 2023). However, since parasites can mediate host resilience to environmental stress in ways that vary across ecological and spatial contexts, it is crucial to clarify how parasitic infection modifies the limits of thermal tolerance and physiological responses of hosts to environmental changes.

In the intertidal zone, the blue mussel is a prominent and essential ecosystem engineer, constructing complex biogenic reefs that create three‐dimensional microhabitats with a rich associated fauna. During emersion, the body temperature of blue mussels can rise well above ambient air temperatures, rendering them sensitive to ongoing climate warming (Helmuth, 1999). Blue mussels serve as an intermediate host for several trematode parasites, including *Himasthla* spp., *Renicola parvicaudatus* and *Psilostomum brevicolle* (De Montaudouin et al., 2000, 2009; Thieltges & Reise, 2006a), and a recent study found that infection levels greater than 250 parasites increased survival following exposure to high air temperatures (Selbach et al., 2020). The study further proposed that a parasite‐induced upregulation of heat shock proteins (Hsps) potentially mediated the impacts of heat stress (Selbach et al., 2020). High infection levels have previously been shown to induce *hsp* expression in fish, crustaceans and molluscs, due to parasite‐induced tissue damage, oxidative stress and immune activation (Evensen et al., 2023; Frank et al., 2013; Liu et al., 2014; Xu & Faisal, 2009). Hsps are highly conserved molecules across species, from bacteria to humans (Hazra et al., 2023; Lindquist & Craig, 1988), and their structure and function have remained largely unchanged throughout evolution, highlighting their essential roles in maintaining and restoring cellular processes (Hu et al., 2022; Krebs & Feder, 1997). The prominent Hsp families, Hsp24, Hsp70 and Hsp90, are of particular interest in thermal biology as they act as molecular chaperones, helping to maintain cellular functioning by assisting in protein folding, transport, inflammation regulation and protein degradation, especially under stressful conditions (Jeyachandran et al., 2023; Salahuddin, 2015). Thus, parasite‐induced elevated levels of these Hsps could improve resilience to acute heat stress by acting as a form of preconditioning factor, effectively priming the host to withstand acute increases in temperature. Although parasite‐induced Hsp levels have been suggested to enhance survival under different stress conditions in both oysters and isopods (Encomio & Chu, 2007; Sasaki et al., 2023), the actual expression levels of Hsps and their correlation with enhanced survival rates in parasite‐infected blue mussels following thermal stress are yet to be evaluated. Therefore, by investigating whether trematode infection influences resilience to heat stress in intertidal blue mussels, and exploring the potential physiological mechanisms underlying this effect, this study tests the hypotheses that (i) high trematode infection levels increase resilience to acute heat stress during emersion and (ii) the expression of *hsp24*, *hsp70* and *hsp90* increases in response to elevated parasite infection levels.

## 2 MATERIALS AND METHODS

### 2.1 Sampling and holding conditions

Subtidal blue mussels *Mytilus edulis* with a mean shell length of 37.5 ± 2.6 (SD) were obtained from cultivation lines suspended in the water column (0–2 m depth) at the aquaculture farm ‘Havhaven’ in Ebeltoft Vig, Denmark (56°11′51.1″ N 10°40′22.1″ E), on 5 September 2024. Rope‐growing blue mussels are generally protected against trematode infection because the intermediate host, the common periwinkle *Littorina littorea*, is absent in the water column (Buck et al., 2005). A total of 182 mussels were collected for the experiment. To estimate the prevalence of parasites in the mussel population, 12 individuals were randomly selected and dissected to detect trematode infections prior to the experiment. Although a 50% infection rate was observed, the low parasite load (<5 per individual) can be considered negligible. All mussels were brought to Aarhus University, Denmark, where they were placed in 16 containers (Condi bucket 1.15 L, Condi Aps) with 10 mussels in each, an air supply and 700 mL of artificial saltwater (Instant Ocean®, Sea salt, Aquarium systems), at a salinity of 27. The containers were placed in a 17°C climate room, and the mussels were fed 0.4 mL of Instant Algae Shellfish Diet 1800 (Reed Mariculture) every other day. During the infection period, mussels were fed daily to increase infection success by stimulating filtration. The water was changed daily, and the mussels were kept under a diel cycle of 8‐h light:16‐h dark.

Common periwinkles infected with parasites were collected intertidally on 7 September 2024, during low tide near Nappedam Harbour, Denmark (56°16′35.3″ N 10°30′02.7″ E). The snails were kept dry in a bucket at 17°C overnight.

Ethics approval was not required for this study, as it involved invertebrate species for which approval from an animal ethics committee is not required under applicable regulations.

### 2.2 Experimental parasite infection

We aimed to establish two different levels of blue mussel trematode infections: low (~50 metacercariae per individual mussel) and high (~150 metacercariae per individual mussel). The shedding of cercariae from common periwinkles was induced by transferring the snails individually to separate containers. Each container contained 155 mL of artificial saltwater with a salinity of 27. The snails were placed in a south‐facing window, exposed to direct sunlight to maintain a temperature of approximately 25°C. During each round of infection, 96 snails were arranged in the window to ensure that at least 32 individuals successfully released cercariae. We used a snail‐to‐mussel ratio of 4:10 for releasing cercariae. After 2 h, the water was examined for free‐swimming cercariae, and containers without cercariae were discarded. Following the release of cercariae from common periwinkles, mussels were quickly infected with freshly shed cercariae from the trematode parasite *Himasthla elongata* to ensure that the cercariae were no more than 2 h old. To optimise the infection process, mussels were kept in water containing cercaria and food until the next tidal cycle (22 h). To reach high infection levels, mussels were subjected to four rounds of infection over a span of 4 days, while low infection levels were obtained by subjecting mussels to a single round of infection. Specifically, high infection was induced over four consecutive days (Days 2–5), while low infection occurred only on Day 5. These timing parameters were selected to facilitate metacercaria formation prior to dissection.

### 2.3 Experimental design

All mussels were exposed to a simulated 2‐h low tide once daily. During a seven‐day acclimation period, all mussels were removed from the buckets and placed on plates in the climate room at 17°C. Individuals that died during this period were removed from the experiment. After acclimation, mussels were divided into two experimental groups: one for the survival experiment and one for gene expression analysis (Figure S1).

For the survival experiment, mussels of the two infection levels (low and high) were exposed to three different air temperatures: 31°C (*n* = 20 for each infection level), 33°C (*n* = 20 for each infection level) and 35°C (*n* = 20 for each infection level) by placing them on plates in three different climate chambers (Memmert ICH110, Memmert GmbH, Schwabach, Germany) for 10 consecutive days, resulting in six different treatments. After each tidal cycle, all mussels were returned to their 700‐mL buckets (salinity = 27) for 22 h at 17°C until the next simulated low tide. During daylight hours, mussels were checked for mortality approximately every 2 h. Mortality was assessed by checking whether mussels could withdraw their siphon or close their shell, and the time of death was noted. 24 h after the final temperature exposure, the mortality of the mussels was assessed for the last time, and the time of death was recorded. After removing a dead mussel, the shell was measured with a vernier calliper to the nearest 0.05 mm, and the infection intensity was quantified. To quantify the intensity of *H. elongata* infection, soft tissue was dissected, tightly squeezed between two glass plates and the metacercariae were counted using a stereomicroscope (Leica MZ12.5 Ergo stereo microscope).

For the gene expression analysis, 40 mussels were exposed to air temperatures of 17°C (*n* = 10 for each infection level) or 33°C (*n* = 10 for each infection level) for 2 h over 3 days, resulting in four different treatments. The 17°C treatment was selected for the gene expression as a control, representing a conservative, non‐stressful summer sea temperature in temperate regions (Thyrring et al., 2015). Whereas 33°C was selected based on pilot trials conducted by the authors on mussels from the same population prior to this study. Exposure to 33°C induced clear signs of thermal stress, with mussels observed to remain open but show little or no ventilation activity, while still responding to tactile stimulation. Mortality was first observed after the fourth temperature exposure. This temperature and exposure duration, therefore, allowed the induction of thermal stress while ensuring that individuals remained alive at the time of sampling, which was essential for quantifying stress‐induced gene expression rather than responses associated with mortality. Mussels exposed to 17°C were placed on plates in the climate room, while mussels exposed to 33°C were placed in the climate chamber. Immediately after the third temperature exposure, shell length was measured, and foot tissue was removed and snap‐frozen using liquid nitrogen. Foot tissue samples were stored at −80°C.

### 2.4 Analysis of gene expression

One‐eighth of each foot tissue sample (<10 μg ww) was obtained from the 40 mussels used for gene expression analysis. Qiagen TissueLyser was used to homogenise the samples, after which RNA extraction was completed using a MicroEluete® Total RNA Kit I (Omega Bio‐Tek), according to the manufacturer's instructions. A QubitMT RNA BR Assay Kit and a Qubit® 2.0 Fluorometer were used to quantify the total amount of RNA. cDNA was synthesised from 1000 ng of total RNA from each sample using the Omniscript reverse transcriptase kit (Qiagen), following the Quick‐Start protocol (Qiagen). The cDNA eluate was then diluted to a concentration of 4 ng/μL and stored at −20°C. Real‐time qPCR was performed on a Stratagene MxPro—MX3005P qPCR system (AH Diagnostics), as described by Waagner et al. (2013). The following genes were tested: *hsp70*, *hsp90* and *hsp24* (Table 1).

**TABLE 1:** Genes tested in the gene expression analysis with their expected target responses and primer sequences.

Gene	Response	Primer sequence	References
hsp24 Heat shock protein 24 kDa	Temperature	Forward primer: 5′‐AGATGACAGTTCCACGGTCTG Reverse primer: 5′‐TGCCCGGATAGTAAGATTGCC	Lockwood et al. (2010)
hsp70 Heat shock protein 70 kDa	Temperature and infection	Forward primer: 5′‐GGGTAGAGAGGACAACTGATGC Reverse primer: 5′‐TTCGCTCCTTTCTGTGGGAG	Evensen et al. (2023), Li et al. (2013), Liu et al. (2014) and Xu and Faisal (2009)
hsp90 Heat shock protein 90 kDa	Temperature	Forward primer: 5′‐TCAGCAACAAGGTAAGCGGA Reverse primer: 5′‐TCATGGAGGCTCTTCAAGCTG	Li et al. (2013)

*Note*: Primer lengths are 20–21 bp.

To evaluate data quality, amplification plots and dissociation curves were examined for signs of poor PCR runs and unspecific amplification. The low‐quality runs were subsequently excluded, and the remaining data were converted to linear *R*_0_ values using DART‐PCRv1.0 (Peirson, 2003), resulting in the following number of replicates from the temperature 17°C: *n*_Low_ = 4, *n*_High_ = 8, and for the temperature 33°C, the number of replicates was: *n*_Low_ = 7, *n*_High_ = 8. Gene expression data were normalised with NORMA‐Gene (Heckmann et al., 2011).

### 2.5 Data analysis

Data analyses and graphical visualisations were performed in R (R Core Team, 2025) using the packages ggplot2 (Wickham, 2016), car (Fox & Weisberg, 2019), survival (Therneau, 2023), survminer (Kassambara et al., 2024) and ggpubr (Kassambara, 2023). A Mann–Whitney *U* test was performed to investigate the difference in the number of parasites between the two infection levels (low and high). Differences in mussel length between the eight treatments were assessed using a one‐way ANOVA. Analysis did not reveal statistically significant differences in mussel lengths between treatments (*F*_5,130_ = 2.181, *p* = 0.06). As a result, the length of the mussels was excluded from subsequent analyses.

A generalised linear model (GLM) with a binomial distribution and a logit‐like function was used to assess the effects of temperature and infection level on mussel survival after the experimental period of 264 h. Temperature (31°C, 33°C and 35°C) and degree of infection (low and high) were treated as factors. Model assumptions, including homogeneity and appropriate model fit, were assessed, along with the interpretation of the model. To evaluate the GLM, three likelihood ratio tests were performed. A Cox proportional hazards model was applied to evaluate the effect of infection on mortality rates. This model included the infection level as the independent variable and was fitted only to the 35°C treatment group. The proportional hazards assumption was verified before interpreting the results.

A two‐way ANOVA was used to assess whether the gene expression levels of *hsp24*, *hsp70* and *hsp90* differed significantly as a function of temperature and infection level, both treated as factors. A logarithmic transformation was applied to the gene expression data to ensure normal distribution. This transformation was deemed appropriate based on visual assessments of the QQ plots and the residual distributions. A post hoc pairwise (Tukey's HSD) test was used to compare significant treatment effects. A significance level of *p* < 0.05 was used for all statistical tests.

## 3 RESULTS

### 3.1 Parasite infection

Post‐experimental dissection revealed a significant difference in the number of metacercariae between mussels from the low (55.3 ± 35.6 SD) and the high (148.6 ± 78.2 SD) infection levels (*U* = 3006, *p* < 0.001). In the low infection level, the number of metacercariae in a mussel ranged from *3* to *152*, whereas in the high infection level, the number ranged from *41* to *397* (Figure S2).

### 3.2 Survival

**FIGURE 1:**
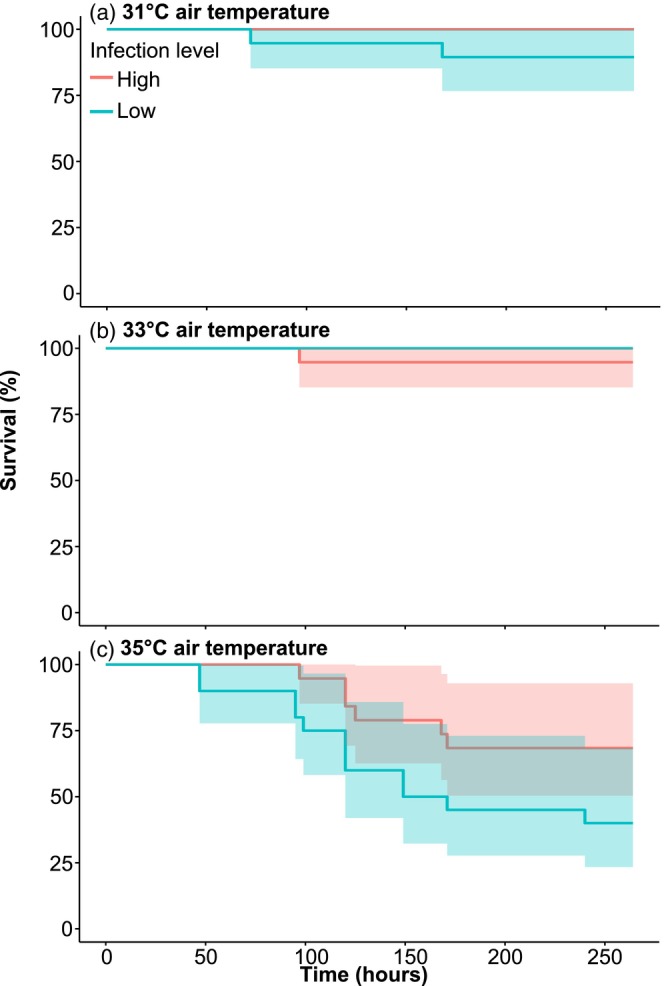
Survival (%) in blue mussels *Mytilus edulis* as a function of time (hours). Mussels were divided into two infections degrees low and high, and exposed to three temperatures during emersion (a: 31°C, b: 33°C and c: 35°C). The shaded area represents the confidence intervals from the survival test.

Air temperature had a significant effect on mussel survival ($\chi^{2}\left(2\right)=31.49$, *p* < 0.001), and although not statistically significant, mussels with a high infection level showed a trend towards increased survival at 35°C ($\chi^{2}\left(1\right)=3.52$, *p* = 0.06) (Figure 1). After 10 emersion cycles, survival in mussels with a high infection level decreased from 100% at 31°C to 68.42% at 35°C, whereas survival in the low infection group decreased from 94.4% to 40% (Figure 1). At 35°C, mussels with a low infection level started to die approximately 50 h after the first air exposure and had an estimated 2.48‐fold increase in mortality risk compared to mussels with a high infection level (HR = 2.48, 95% CI: 0.93–6.61, *p* = 0.07) (Figure 1c, Table S1).

### 3.3 Gene expression

**FIGURE 2:**
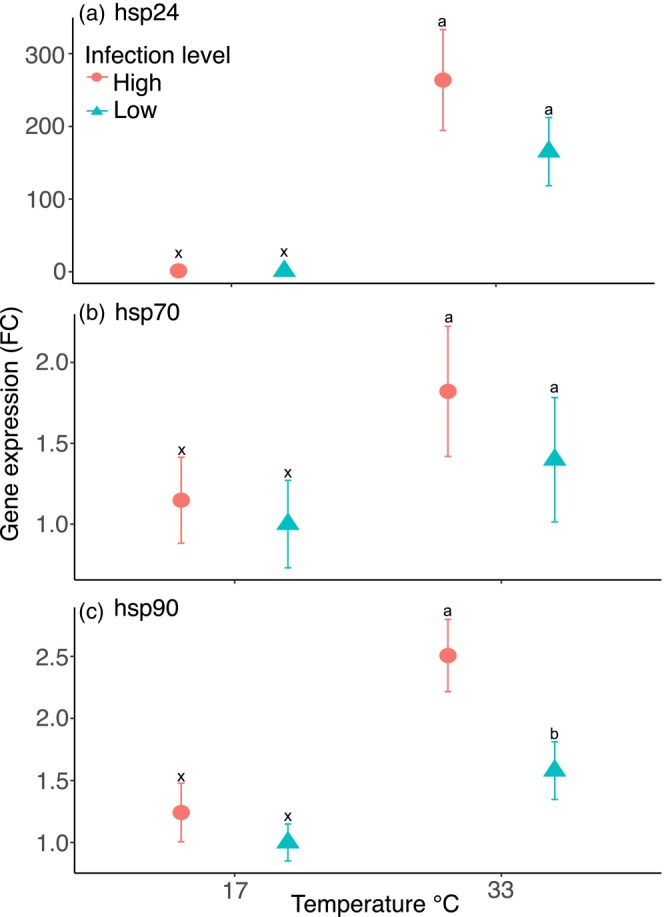
Change in gene expression (fold change, FC) (± SE) of (a) *hsp24*, (b) *hsp70* and (c) *hsp90* in *Mytilus edulis* across two temperatures (17°C and 33°C) and two infection degrees (low and high). Gene expression is log transformed and expressed as fold change (FC) relative to the low infection degree at 17°C; error bars signify standard error. Different letters (33°C) and symbols (17°C) indicate statistical differences between the infection degrees.

The gene expression of all three heat shock proteins (*hsp24*, *hsp70* and *hsp90*) was strongly influenced by temperature, whereas infection intensity had minor or gene‐specific effects (Table S2). *Hsp24* expression increased markedly at 33°C compared to 17°C for both infection levels (Tukey's HSD, *p* < 0.001), with a near‐significant difference between infection levels (Tukey's HSD, *p* = 0.069) (Figure 2a). The expression of *hsp70* was also upregulated at 33°C (Tukey's HSD, *p* = 0.019) (Figure 2b), and *hsp90* expression increased with both increasing temperature (Tukey's HSD, *p* < 0.001), and with increasing infection level (Tukey's HSD, *p* = 0.034), with the highest expression found in high infected mussels after exposure to 33°C (Figure 2c).

## 4 DISCUSSION

Global warming is increasing the frequency and intensity of extreme weather events, posing a major challenge for ectothermic species globally. Increasing temperatures have further been shown to enhance the infection success of the trematode *Himasthla elongata* in mussels (Díaz‐Morales et al., 2022), indicating that interactions between thermal stress and parasitism may intensify in future. The aim of this study was to determine whether parasite infections alter the heat shock response and thermal tolerance of blue mussels. To test this, we examined how parasite infections affect *hsp* expression and survival in blue mussels exposed to elevated air temperatures. Our results show that parasite‐infected mussels exposed to 33°C exhibited an upregulation of *hsp90* and a similar but non‐significant pattern for *hsp24*. High infection levels also exhibited a tendency to increase survival rates in mussels exposed to elevated air temperatures, suggesting that parasite‐induced stress responses might increase resilience to heat stress during low‐tide conditions.

Parasitism is a ubiquitous biological factor that is often overlooked when considering the effects of warming on species distribution, performance and survival (Chrétien et al., 2023). However, our results demonstrate that parasites have the potential to modify the limits of thermal tolerance and physiological responses of hosts to heat stress. Similar to trematodes, endolithic microbes demonstrate that biotic interactions can mediate host resilience to environmental stress in ways that vary across ecological and spatial contexts. For instance, infections by shell‐degrading endoliths, which erode the dark periostracum and expose the lighter underlying shell, can reduce thermal stress by decreasing light absorption, and increased survival has been observed in blue mussels hosting endolithic symbionts following heat waves (Gehman & Harley, 2019; Lourenço et al., 2017; Zardi et al., 2016, 2021). In contrast to our results, De Bonville et al. (2024) showed that trematode infections reduced survival of pumpkinseed sunfish *Lepomis gibbosus* following a simulated heat wave, demonstrating the complex context‐dependent outcomes of parasite infections.

The host response to infection is also influenced by parasite load, with stronger responses observed in hosts that are highly infected (Auld et al., 2012; Cornet & Sorci, 2010; Sistermans et al., 2023). In our study, the intensity of infection did not have a statistically significant effect on survival, contradicting the results of a previous study showing an increased survival rate of more than 10% in blue mussels (*M. edulis*) infected with >250 trematodes after exposure to 35°C (Selbach et al., 2020). The contradicting results may be related to the relatively low infection levels used in our experiment (55.3 metacercariae for low and 148.6 for high infection level), as these lower levels of infection may have obscured the potential survival benefits of high infection under heat stress. Both the number of metacercariae and the number of different parasite species are known to be positively correlated with the size and the age of the host, as well as the density of the first intermediate host of the specific trematode (Desclaux et al., 2006; Richard et al., 2022; Thieltges & Reise, 2006a, 2006b). In natural populations, the average metacercarial infection by a single trematode species is typically between 50 and 200 (Richard et al., 2022), and the intensity of infection, regardless of the trematode species, can reach up to 500 metacercaria per individual (Lauckner, 1984). Thus, >200–300 metacercaria per individual may better represent high infection intensity, and an experiment using higher infection levels and a higher number of mussels could have revealed increased survival under heat stress.

High levels of parasite infection are known to upregulate genes related to the immune system. For example, in the mud crab *Scylla paramamosin*, *hsp90* expression increases after bacterial and viral exposure (Huang et al., 2013), and *hsp* expression has been hypothesised to improve resilience to heat stress in blue mussels (Selbach et al., 2020). Our findings support this hypothesis, as *hsp90* expression was significantly upregulated, and hsp24 showed a weak, non‐significant upregulation in highly infected mussels exposed to 33°C. Thus, the results suggest that parasite‐induced *hsp* expression may contribute to improved thermal tolerance, but more studies are necessary to understand the role of *hsp* in parasite‐infected species, especially considering that the regulation of *hsp* genes is complex and context‐dependent, varying by species, gene variant and stage of infection (Kamel et al., 2019). One mechanism behind parasite‐induced resilience to acute heat stress could be the constitutive frontloading of chaperone genes (such as *hsps*), which play a key role in protecting against environmental stress.

Frontloading is defined as an elevated baseline expression of genes that allows animals to cope with stressful conditions (Collins et al., 2021). Exposure to sublethal stress can prime organisms to maintain an elevated stress protein level. For example, sublethal heat exposure has been shown to induce metabolic changes, such as increased TCA cycle activity and osmolyte accumulation, improving thermal tolerance in the Mediterranean mussel *Mytilus galloprovincialis* (Georgoulis et al., 2022). Frontloading has also been observed in sand shrimps *Echinogammarus marinus* (Collins et al., 2021) and in chimeric coral colonies (Vidal‐Dupiol et al., 2022), where it enhances resilience to multiple stressors. However, whether frontloading is genetically fixed or plastically induced by prior environmental stressors, such as parasitic infection, remains an open question. Understanding whether parasite infections influence the frontloading of stress‐related genes requires broader transcriptomic approaches, such as RNA‐seq, rather than targeted analyses based on primers for candidate genes as used here. Targeted primers are effective tools to measure change in expression levels, but they restrict the detection of other gene family members, which may be important stress regulators. For example, the Hspa12 gene family is a distant member of the Hsp70 family, and it has been suggested that the expression of the Hspa12 genes has been selected in bivalves as an adaptation to their exposure to a complex of biotic and abiotic stresses (Hu et al., 2022; Kim et al., 2024). In *M. edulis* specifically, Hspa12 has been proposed as a key stress regulator during exposure to heat, but its potential role in parasite‐induced resilience to heat stress requires more experiments and analysis of the gene network (Clark et al., 2021).

Although potentially beneficial, elevated gene expression comes with metabolic costs. Genes utilise substantial amounts of ATP, representing an energy investment for the organism (Krebs & Feder, 1997; Kühnhold et al., 2019). Consequently, improved stress tolerance, whether induced by parasite infection or other environmental factors, is likely associated with physiological trade‐offs, as the energy required to maintain elevated stress responses can be diverted from other vital processes, such as growth, reproduction, or immune function (Sørensen et al., 2003). For example, in *Drosophila melanogaster*, overexpression of Hsp70 has been linked to reduced fecundity and lower egg hatching rates, illustrating the reproductive costs of elevated Hsp activity (Silbermann & Tatar, 2000). These costs may influence the overall condition of the organism, with potential consequences for population dynamics in intertidal species. In parasite‐rich and thermally variable environments, such trade‐offs could affect long‐term survival and reproductive success, especially under ongoing climate change that intensifies both thermal stress and parasitic pressures (Tomanek, 2010).

The relationship between parasitism and heat resilience is clearly complex. Blue mussels can acclimate to fluctuating temperatures (Widdows, 1976), and parasite‐induced frontloading could build on this capacity, though more experiments are needed to confirm this. Continuous warming may enhance the infection success of parasites (Díaz‐Morales et al., 2022), and under increasingly frequent and intense heatwaves, parasite‐induced stress responses could shift from being a cost to becoming a critical mechanism for host survival, potentially moving the relationship from parasitism towards mutualism. Although our findings demonstrate that parasite‐induced *hsp* expression can enhance thermal resilience during emersion, the magnitude and consistency of this effect may differ in natural populations, where temperature, humidity, solar radiation and exposure duration interact with multiple biotic stressors (Helmuth et al., 2016; Moreira et al., 2023). This variability can increase or mask the influence of parasitic infection, and the relative importance of trematode infection under naturally variable conditions remains to be quantified.

## AUTHOR CONTRIBUTIONS

All authors conceived the ideas and designed the methodology; Annika Greve collected the data; Annika Greve and Jakob Thyrring analysed the survival data; Annika Greve and Jesper G. Sørensen analysed the qPCR data; Annika Greve and Jakob Thyrring led the writing of the manuscript. All authors contributed critically to the drafts and gave final approval for publication.

## CONFLICT OF INTEREST STATEMENT

No competing interests are declared by the authors.

## STATEMENT ON INCLUSION

Our study was a laboratory‐based experiment conducted in Denmark using blue mussels sourced from a local aquaculture facility, ‘Havhaven’, in Ebeltoft Vig. All authors are based in Denmark and were fully involved in the study design, execution, and interpretation of results. While no fieldwork was conducted, we engaged with Havhaven both in the sourcing of experimental animals and in sharing the results of our parasite screening, providing them with insight into baseline parasite levels in their mussels. This exchange contributed to local awareness of parasite presence in mussels intended for consumption.

## Supporting information


**Figure S1:** Number of mussels per treatment and type of Analysis.
**Figure S2:** The number of metacercariae per Individual blue mussel (*Mytilus edulis*) according to the two infection levels, Low and High.
**Table S1:** Hazard ratios (HR) and 95% confidence intervals (CI) were estimated using Cox models.
**Table S2:** Results of the two‐way ANOVA for the expression of genes (hsp70, hsp90 and hsp24) in blue mussels (*Mytilus edulis*).

## Data Availability

Data available from the Zenodo Digital Repository: https://doi.org/10.5281/zenodo.17201358 (Greve et al., 2025).
