# Supplementary material for: Trematode infection buffers heat stress in blue mussels Mytilus edulis: The role of heat shock proteins
Source: J Anim Ecol. 2026 Jan 26;95(4):648–57. doi: 10.1111/1365-2656.70220 (PMC13039247; doi:10.1111/1365-2656.70220)
Supplement: Supplementary file 1 — Figure S1: Number of mussels per treatment and type of Analysis. Figure S2: The number of metacercariae per Individual blue mussel (Mytilus edulis) according to the two infection levels, Low and High. Table S1: Hazard ratios (HR) and 95% confidence intervals (CI) were estimated using Cox models. Table S2: Results of the two‐way ANOVA for the expression of genes (hsp70, hsp90 and hsp24) in blue mussels (Mytilus edulis). [file JANE-95-648-s001.docx]

Supplementary Information for

Trematode Infection Buffers Heat Stress in Blue Mussels (*Mytilus edulis*): The Role of Heat Shock Proteins

Annika Greve ^a, *^, Jesper G. Sørensen ^b^, Mikael K. Sejr ^a^, Jakob Thyrring ^a^

^a^ Department of Ecoscience, Marine Ecology, Aarhus University, 8000 Aarhus C, Denmark

^b^ Department of Biology, Section for Genetics, Ecology and Evolution, Aarhus University, 8000 Aarhus C, Denmark

^*^Correspondence to AG: E-mail: [ang@ecos.au.dk](mailto:ang@ecos.au.dk) Adress: C. F. Møllers Allé 3, 1131-621, 8000 Aarhus C, Denmark.

Content:

Figure S1: Number of mussels per treatment and type of Analysis.

Figure S2: The number of metacercariae per Individual blue mussel (*Mytilus edulis*) according to the two infection levels, Low and High.

Table S1: Hazard ratios (HR) and 95% confidence intervals (CI) were estimated using Cox models.

Table S2: Table S2: Results of the two-way ANOVA for the expression of genes (hsp70, hsp90 and hsp24) in blue mussels (Mytilus edulis).


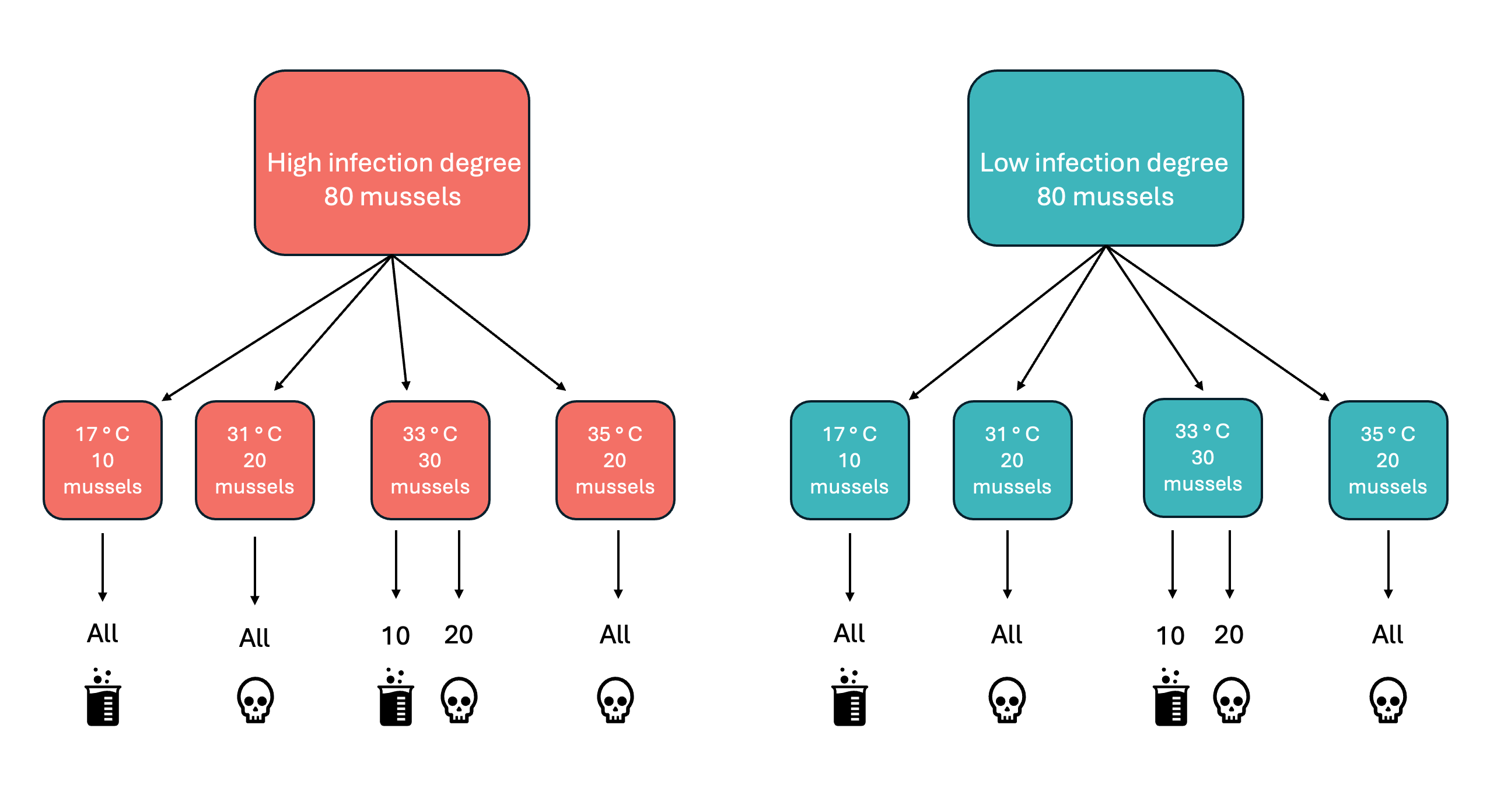
Figure S1: Number of mussels per temperature treatment, infection degree, and experimental endpoint, gene expression (Griffin beaker) or survival test (Skull).

Figure S2: The number of metacercariae per Individual blue mussel (*Mytilus edulis*) according to the two infection levels, Low and High. X signifies mean.

Table S1: Hazard ratios (HR) and 95% confidence intervals (CI) were estimated using Cox models. For treatments with only 1–2 events, estimates were unstable (coefficients ±∞, CIs ∞) and are reported for transparency. At 35 °C, sufficient events allowed reliable HR estimation.

| Temperature (° C) | Events (n) | HR (95 % CI) for infection degree | p-value | Notes |
| --- | --- | --- | --- | --- |
| 31° C | 2 | 1.68 X 10^-9^ (0-$\infty$) | 0.999 | Only one mortality; model unstable |
| 33 ° C | 1 | 6.45 X 10^8^ (0-$\infty$) | 0.999 | Only two mortalities; model unstable |
| 35 ° C | 18 | 2.48 (0.93-6.61) | 0.07 | Model converged |

Table S2: Results of the two-way ANOVA for the expression of genes (*hsp24*, *hsp70* and *hsp90*) in blue mussels (*Mytilus edulis*). The blue mussels were exposed to four different treatments consisting of two temperature (17 ° C and 33 ° C) and two infection levels (low and high). Data has been normalized and logarithmic transformed.

|  | df | Sum sq | F value | Pr(>F) |
| --- | --- | --- | --- | --- |
| *hsp24* |  |  |  |  |
| Temperature | 1 | 195.28 | 863.14 | <0.001 |
| Infection level | 1 | 0.88 | 3.86 | 0.061 |
| Temperature: Infection level | 1 | 0.16 | 0.72 | 0.405 |
| Residuals | 24 | 5.43 |  |  |
| *hsp70* |  |  |  |  |
| Temperature | 1 | 1.11 | 6.35 | 0.019 |
| Infection level | 1 | 0.43 | 2.49 | 0.128 |
| Temperature: Infection level | 1 | 0.04 | 0.24 | 0.627 |
| Residuals | 24 | 4.177 |  |  |
| *hsp90* |  |  |  |  |
| Temperature | 1 | 2.63 | 19.45 | <0.001 |
| Infection level | 1 | 0.70 | 5.20 | 0.032 |
| Temperature: Infection level | 1 | 0.21 | 1.59 | 0.220 |
| Residuals | 23 | 3.1068 |  |  |
